# Detection of *BCOR* gene rearrangement in Ewing-like sarcoma: an important diagnostic tool

**DOI:** 10.1186/s13000-021-01114-2

**Published:** 2021-06-08

**Authors:** Lan Li, Ming Zhang, Shaoyu Chen, Xiaoqi Sun, Hairong Xu, Lina Li, Tingting Zhang, Xiaoyuan Huang, Hongtao Ye, Yi Ding

**Affiliations:** 1 Department of Pathology, Beijing Jishuitan Hospital, The Fourth Medical College of Peking University, Beijing, People’s Republic of China; 2 Guangzhou LBP Medicine Science & Technology Co., Ltd, Guangzhou, People’s Republic of China; 3 Department of Orthopedic Oncology Surgery, Beijing Jishuitan Hospital, The Fourth Medical College of Peking University, Beijing, People’s Republic of China; 4 Department of Histopathology, Royal National Orthopaedic Hospital, Stanmore, UK; 5 Department of Pathology, Affiliated Hospital of Guilin Medical University, Guilin, People’s Republic of China

**Keywords:** Undifferentiated small round cell sarcoma, *BCOR*, FISH, RT-PCR

## Abstract

**Background:**

*BCOR-CCNB3* sarcoma (BCS) is a group of undifferentiated small round cell sarcomas harboring the *BCOR* gene rearrangement which shares morphology with the Ewing sarcoma family as well as other malignant round blue cell tumors, thus making them difficult to diagnose. The aim of this study was to explore the role of molecular techniques in the diagnosis of BCS.

**Methods:**

Twenty-three cases of *EWSR1* rearrangement-negative undifferentiated small round cell sarcomas (Ewing-like sarcoma) were analyzed for the presence of *BCOR* gene rearrangement by Fluorescence in situ hybridization (FISH) and Reverse Transcription -Polymerase Chain Reaction (RT-PCR). The clinicopathological features of the positive cases were also reviewed. Fifteen additional cases were used as negative controls.

**Results:**

Eight cases were found with *BCOR* gene rearrangement by FISH and reappraised as BCS. The patients ranged in age from 8 to 20 years old, with a male predominance (M:F = 6:2). All tumors were located in the lower extremities. The tumor locations were more common in bone (*n* = 6) than deep soft tissue (*n* = 2). Histologically, 7 of 8 tumors were predominately composed of spindle or ovoid cells. The tumor cells were usually arranged in solid hypercellular sheets without a distinct architectural pattern. IHC showed expression of TLE1 (100%), CCNB3 (88%), BCOR (71%). RT-PCR for *BCOR-CCNB3* fusion transcript was positive in 7 of 8 cases. Pre-operative chemotherapy resulted in eradication of tumors in 5 patients after a follow-up of 7 to 42 months.

**Conclusions:**

Efficient diagnosis of *BCOR* rearranged sarcomas is achieved by the using a combination of FISH and RT-PCR assays.

**Supplementary Information:**

The online version contains supplementary material available at 10.1186/s13000-021-01114-2.

## Background

*BCOR-CCNB3* sarcomas (BCS) were first identified in 2012 from a series of undifferentiated round cell sarcomas lacking known genetic alterations such as *EWSR1* gene rearrangement [1]. Recently, several studies have demonstrated that similar to the epidemiology of Ewing sarcoma, BCS occurs predominantly in adolescents and young adults [2–7]. Although tumors harboring the *BCOR-CCNB3* fusion appear to share some clinical and morphological overlap with the Ewing family of tumors, sequencing analysis has shown that the rearrangement involves a paracentric inversion on the short arm of chromosome X, resulting in the fusion of two genes *BCOR* and *CCNB3* and resulting in the expression of CCNB3. Moreover, by gene expression profiling, BCS appear distinct from Ewing sarcoma (ES) [2]. We investigated the prevalence of the *BCOR-CCNB3* fusion in pediatric and adult undifferentiated small round cell sarcomas, using a combination of FISH and RT-PCR and report on the clinical and histopathological features of eight patients with sarcomas harboring this fusion gene.

## Methods

### Specimens

Twenty-three cases of *EWSR1* rearrangement-negative undifferentiated small round cell sarcomas (Ewing-like sarcoma) were analyzed for the presence of the *BCOR* gene rearrangement. All of the cases were retrieved from the archives of Department of Pathology, Beijing Jishuitan Hospital, The Fourth Medical College of Peking University. All paraffin blocks selected were rich of tumors without decalcification. Fiften cases including 7 PNET/Ewing sarcomas, 5 synovial sarcomas, 1 osteosarcoma and 2 malignant peripheral nerve sheath tumors were selected as negative controls. Representative paraffin-embedded material and haematoxylin and eosin-stained slides were reviewed for all cases. The study complied with local ethical standards. The study protocol was approved by the ethics committee at the Beijing Jishuitan Hospital, China.

Mitotic figures were counted in 10 consecutive high-power fields (1 HPF = 0.238 mm^2^) in highly proliferative ‘hot spot’ areas.

### Immunohistochemistry (IHC)

Immunohistochemistry was performed using antibodies to CD99 (O13, monoclonal, ready to use, Roche, Basel, Switzerland), Fli-1 (G146–22, monoclonal, 1:50; OriGene, Maryland, United States), CCNB3 (polyclonal, 1:300; Sigma-Aldrich, St. Louis, MO), BCOR (C-10, monoclonal, 1:100; OriGene, Maryland, United States), DUX4 (P4H2, monoclonal, 1:250; Thermo Fisher Scientific, Massachusetts, United States), NKX2.2 (EP336, monoclonal, 1:100; Origene, Maryland, United States), WT-1 (6F-H2, monoclonal, 1:100; Dako, Glostrup, Denmark), calretinin (polyclonal, 1:100; OriGene, Maryland, United States), MUC4 (8G7, monoclonal, 1:50; OriGene, Maryland, United States), TLE1 (UMAB253, monoclonal, 1:100; OriGene, Maryland, United States), EMA (GP1.4, monoclonal, 1:100; OriGene, Maryland, United States). Diaminobenzidine was used as a chromogen in all reactions. Positive and negative controls were included in each immunohistochemistry run.

### Fluorescence in situ hybridization (FISH)

FISH was performed using the commercially available *BCOR* dual color break apart probe (Guang Zhou LBP Medicine Science and Technology, Guangzhou, China). In brief, deparaffinized sections were digested with pepsin at 37 °C for 9 mins. Subsequently, the tissue sections and *BCOR* break apart probe were co-denatured at 85 °C for 5 mins and hybridized overnight at 37 °C. Following hybridization, washing was performed. Slides were then counterstained with 4′, 6-diamidino-2-phenylindole (DAPI) and mounted with coverslips. A positive result was obtained when at least 10% of the nuclei analyzed revealed a break apart signal on counting a minimum of 100 consecutive non-overlapping nuclei. Unlike other typical positive patterns of break apart signals, the distance between the green and red signal for *BCOR* rearranged case is a small gap reflective of the underlying paracentric inversion and is usually less than the diameter of two signals. *BCOR* signals were scored by independently two experienced pathologists.

### RNA extraction and reverse transcription (RT)

Two 10 μm or 5–10 10 μm sections were cut from resection or biopsy specimen blocks, respectively, and placed into Eppendorf tubes. RNA was extracted from paraffin-embedded samples using FFPE RNA Isolation Kit (ThermoFisher Scientific, USA). Between 5 and 8 μl of the resulting RNA samples were reverse transcribed using Superscript III First-Strand Synthesis kit (ThermoFisher Scientific, USA) according to the manufacturer’s instructions.

### Conventional polymerase chain reaction (PCR) and sanger sequencing

PCR amplification was performed on duplicate samples of 1 μl aliquots of cDNA with HotStarTaq DNA polymerase (Qiagen, Valencia, USA) using primers (*BCOR* exon 15 – AGGAGCTGTTAGATCTGGTGGA) and *CCNB3* exon 5 –GTGGTTTCTCCATAATGTTTGGT) in order to generate a 171-bp product [3]. A touchdown protocol was used with cycling parameters as follows: 7 min at 95 °C followed by 45 s at 94 °C, 45 s at 66 °C, 1 min 30s at 72 °C which was followed by reducing the annealing temperature by 1 °C each cycle to 57 °C (10 cycles), followed by 30 cycles at 56 °C and finally 5 min at 72 °C. Products were separated through an 8% polyacrylamide gel, stained with ethidium bromide and visualized under UV illumination. The house-keeping gene *G6PD* was used for RNA quality control. Direct Sanger sequencing was performed using BigDye Terminator v3.1 chemistry (Life Technologies) on positive cases.

## Results

### Clinical and histological features

**Fig. 1:**
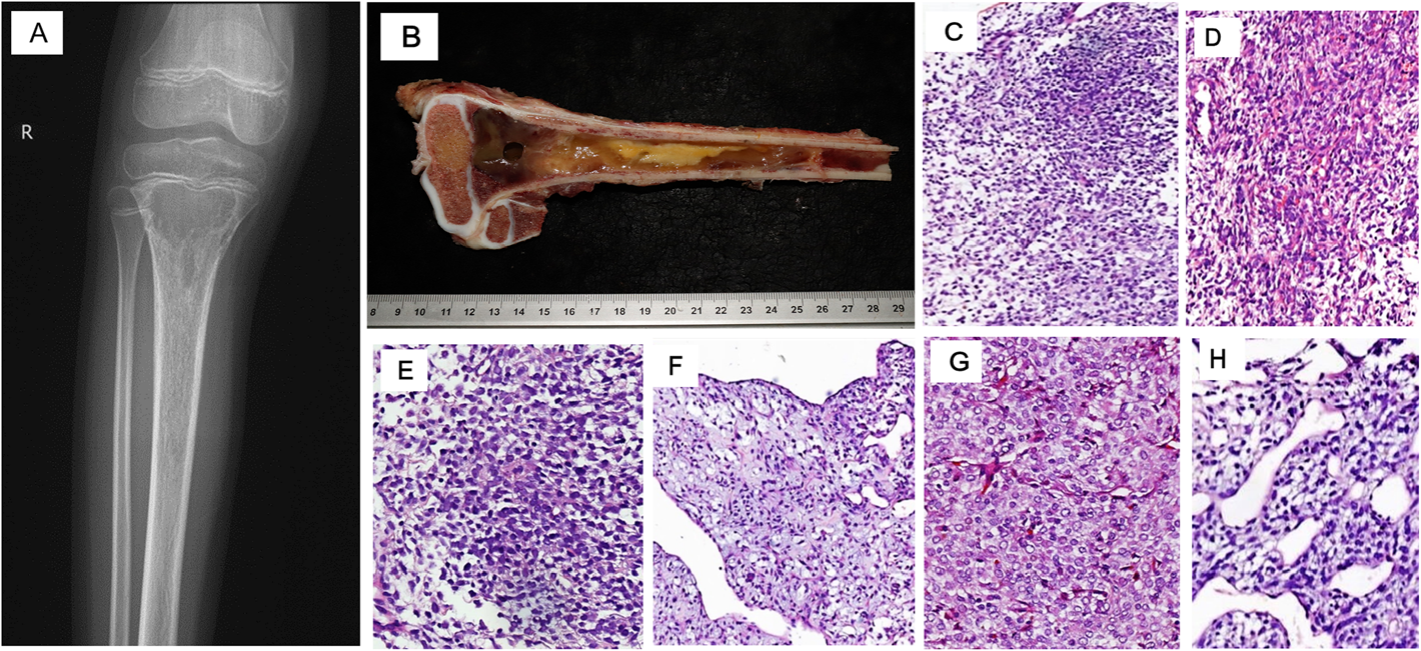
Radiological, macroscopic and histological features of BCS. **A** X-Ray showed a lytic mass with irregular margins in right proximal tibia. **B** Sample after chemotherapy showed grey-yellow or brown tumors in the medullary cavity, some areas had gel appearance. **C** Tumor cells arranged in solid hypercellular sheets without a distinct architectural pattern. **D** Tumor cells arranged in a vague whirling pattern. **E** The tumor cells showed monomorphic, ovoid nuclei, with similar fine chromatin pattern. **F** Hypocellular myxoid areas was focally seen. **G** One case (case 7) was composed predominantly of primitive round cells. **H** One case (case 5) showed markedly perivascular arrangement and cell clustering. Haematoxylin and eosin, original total magnification × 200 (**C**-**H**)

Twenty-three cases of *EWSR1* rearrangement-negative undifferentiated small round cell sarcomas were analyzed by FISH and RT-PCR respectively. Eight of 23 cases were positive for *BCOR* gene rearrangement by FISH analysis (Table 1). Among these 8 cases, 3 tumors were needle core biopsies and 5 were resection samples. The percentage of samples with break-apart signals in this study varied from 21 to 53% of the cells in the cases in which *BCOR* gene rearrangement was found. These cases are considered as *BCOR*-rearranged sarcoma. Seven of 8 cases carrying *BCOR* gene rearrangement were positive for *BCOR-CCNB3* fusion transcript by RT-PCR. Patients with these tumors presented between the ages of 8 and 20, the mean age being 12 years and the tumors were more prevalent in males than females (6 males and 2 females). All primary tumors were located in lower extremities (Table 1). Radiological review showed that most cases presented as lytic masses with irregular margins on plain X-rays (Fig. 1a).

**Table 1:** Clinicopathologic factors in BCS patients

Case	Age/Sex	Location	Size (cm)	BCOR FISH positive	BCOR-CCNB3 RT-PCR positive	neoadjuvant chemotherapy	surgery	Chemotherapy/Radiation (after surgery)	Follow-up (months)	Recurrence and Metastasis (Site)
1	14/M	Calcaneus	5	Yes	Yes	Yes^a^	Yes*	Chemotherapy^a^	46(DOD)	Recurrence & metastasis to lung
2	20/M	Femoral shaft	12	Yes	Yes	Yes^b^	Yes*	Chemotherapy^a^	40(NED)	No
3	8/M	Fibula	NA	Yes	Yes	No	No	Chemotherapy^a^ +Radiation^g^	42(NED)	No
4	10/M	Proximal tibia	16	Yes	Yes	Yes^c^	Yes*	Chemotherapy^d^	29(NED)	No
5	18/M	Distal femur	NA	Yes	Yes	Yes^d^	Yes*	Chemotherapy^d^	22(NED)	No
6	13/F	Leg	NA	Yes	Yes	Not	Yes	Chemotherapy†^a^	9(NED)	No
7	10/M	Leg	NA	Yes	No	Yes†^a^	ND	ND	9(AWD)	No
8	11/F	Proximal femur	9	Yes	Yes	Yes^e^	Yes*	Chemotherapy^f^	7(NED)	No

*M* male, *F* female, *DOD* dead of disease, *Surgery was performed after neoadjuvant chemotherapy, *NED* no evidence of disease, *AWD* alive with disease, *NA* not available, † chemotherapy was receiving when the article was written, *ND* not determined, ^a^ chemotherapy regimens were Vincristine,Oncovin (VCR),Adriamycin (ADR), Cyclophosphamide (CTX),Ifosfamide (TFO) and Etoposide (VP-16), ^b^VP-16 + IFO + Endostar+Methotrexate (MTX) + Cisplatin (DDP) + VCR + ADR + CTX, ^c^ VCR + ADR + CTX + IFO + MTX, ^d^ IFO + MTX + DDP + ADR, ^e^ ADR + MTX + IFO + Apatinib, ^f^ IFO + VP16 + MTX + DDP + VCR + ADR + CTX, ^g^ DT40G/20fx

Macroscopic findings showed grey, brown soft tumor with medium texture, focally translucent in four cases (case 1, 2, 4 and 8). Some areas had a gelatinous appearance (Fig. 1b). Soft tissues infiltration around the tumors were found in 6 of 8 cases.

**Fig. 2:**
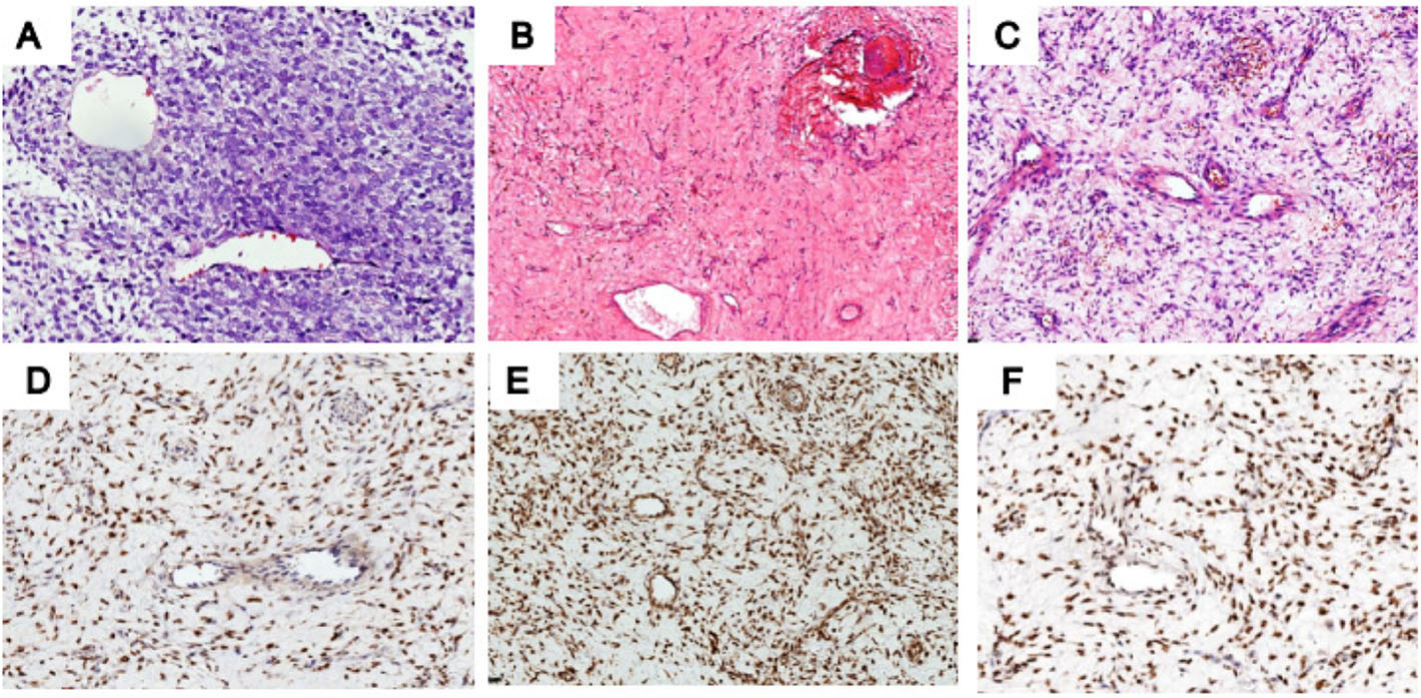
Histological and immunohistochemical features of BCS that received pre-operative chemotherapy. **A** The tumor cells arranged in perivascular pattern (before chemotherapy). **B** The tumors showed good response to chemotherapy, with total replacement of tumor cells by hypocellular loose fibrous tissue. **C** A focal area of scant perivascular tumor cells created a vascular tumor-like appearance. **D** The residual cells after chemotherapy were strong BCOR-positive in the nuclei. **E** The residual cells after chemotherapy were CCNB3 positive. **F** The residual cells after chemotherapy were TLE1 positive. Haematoxylin and eosin, original total magnification × 200 (**A**-**C**). Immunoperoxidase, original total magnification × 200 (**D**-**F**)

Histological assessment revealed that the tumors were composed of monomorphic spindle or ovoid cells often arranged in solid hypercellular sheets without a distinct architectural pattern (Fig. 1c) and less often in a vague whirling pattern (Fig. 1d). The tumors showed variable cellularity and the nuclei demonstrated a finely dispersed chromatin pattern (Fig. 1e), and hypocellular myxoid areas focally (Fig. 1f). Case 7 was composed of predominantly primitive round cells (Fig. 1g). Most of the tumors showed a rich capillary network which was a notable characteristic (Fig. 1g). Case 5 demonstrated a striking perivascular arrangement and cell clustering (Fig. 1h). Only one patient (case 1) showed recurrent and metastatic tumors; both the primary and recurrent specimens were available for analyses. When compared with the primary tumor, the recurrent sarcoma showed a higher degree of pleomorphism with large, highly atypical spindle cells within a fibrotic matrix and hemorrhage and necrosis. Four of the patients (case 2, 4, 5 and 8) that received chemotherapy showed a significant response to chemotherapy (Fig. 2). Two tumors (case 2 and 8) showed a vascular tumor-like appearance (Fig. 2c). Across all 8 cases, the mitotic activity per 10 HPF ranged from 1 to 11 (mean 8).

### FISH

*BCOR* gene rearrangement was detected in 8 cases. The percentage of the positive cells was 21 to 53% (average 38%). The remaining 15 cases of *EWSR1* gene rearrangement- negative undifferentiated small round cell sarcomas (included 2 *CIC* rearrangement sarcomas and 13 undifferentiated small round cell sarcomas) were negative for *BCOR* gene rearrangement. None of the 15 negative control samples revealed the *BCOR* rearrangement (Fig. 4a, Supplementary Figure 1).

### IHC analysis

**Fig. 3:**
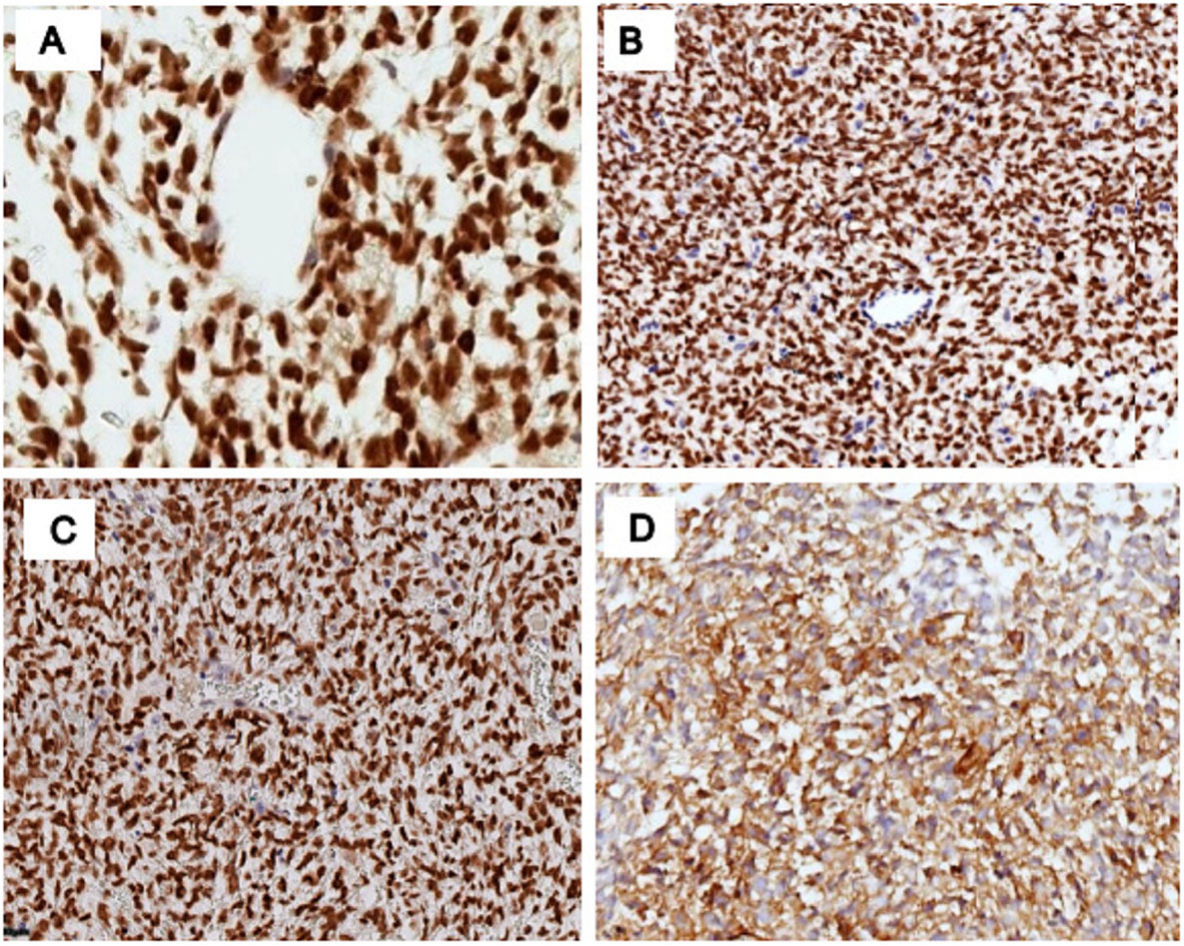
Immunohistochemistry features of BCS. **A** The tumor cells were CCNB3 positive. **B** The tumor cells were BCOR positive. **C** The tumor cells were TLE1 positive. **D** The tumor cells were CD99 focally positive in the cytoplasm. Immunoperoxidase, original total magnification × 200 (**A**-**D**)

Seven of 8 cases showed protein expression of CCNB3 (88%, 7/8 cases), and showed expression of TLE1 (100%, 8/8 cases), BCOR (71%, 5/7 cases), CD99 (13%, 1/8 cases). Fli-1, DUX4, NKX2.2, WT-1, calretinin, MUC4, EMA were all negative in the 8 cases (Table 2) (Fig. 3). The expression of CCNB3, TLE1, BOCR, CD99, Fli-1, DUX4, NKX2.2, WT-1, calretinin, MUC4 and EMA in the 15 cases of *EWSR1* gene rearrangement-negative undifferentiated small round cell sarcomas (Ewing-like sarcoma) and 15 cases of negative controls were included in Table 3.

**Table 2:** IHC characterisations in BCS Patients

Case	CD99	Fli-1	CCNB3	BCOR	DUX4	Nkx2.2	WT-1	calretinin	MUC4	TLE1	EMA
1	Negative	Negative	Positive	Positive	Negative	Negative	Negative	Negative	Negative	Positive	Negative
2	Negative	Negative	Negative	Positive	Negative	Negative	Negative	Negative	Negative	Positive	Negative
3	Negative	Negative	Positive	NA	Negative	Negative	Negative	Negative	Negative	Positive	Negative
4	Positive	Negative	Positive	Positive	Negative	Negative	Negative	Negative	Negative	Positive	Negative
5^a^	Negative	NA	Positive	Negative	Negative	Negative	Negative	NA	NA	Positive	NA
6	Negative	NA	Positive	Negative	Negative	Negative	Negative	NA	NA	Positive	NA
7	Negative	NA	Positive	Positive	Negative	Negative	NA	NA	Negative	Positive	Negative
8*	Negative	Negative	Positive	Positive	Negative	Negative	Negative	NA	Negative	Positive	Negative

^a^IHC was performed on the specimen after neoadjuvant chemotherapy; *NA* not available

**Table 3:** IHC characterisations in the 15 cases of Ewing-like sarcoma and 15 cases of negative controls

	CD99	Fli-1	CCNB3	BCOR	DUX4	Nkx2.2	WT-1	calretinin	MUC4	TLE1	EMA
ELS	13/15 (87%)	6/15 (40%)	0	0	1/12 (8%)	5/15 (33%)	1/11 (9%)	3/10 (30%)	1/10 (10%)	9/15 (60%)	5/11 (45%)
ES	7/7 (100%)	5/7 (71%)	0	0	0	7/7 (100%)	0	0	0	3/7 (43%)	0
SS	3/5 (60%)	3/5 (60%)	0	3/5 (60%)	1/5 (20%)	3/5 (60%)	0	0	0	4/5 (80%)	2/3 (67%)
MPNST	2/2 (100%)	2/2 (100%)	0	2/2 (100%)	0	1/2 (50%)	0	1/10	0	2/2 (100%)	0
OS	1/1 (100%)	1/1 (100%)	1/1 (100%)	1/1 (100%)	0	0	0	0	0	1/1 (100%)	0

*ELS* Ewing-like sarcoma, *ES* Ewing sarcoma, *SS* synovial sarcoma, *MPNST* malignant peripheral nerve sheath tumor, *OS* osteosarcoma

### Detection of *BCOR-CCNB3* fusion transcript by RT-PCR

**Fig. 4:**
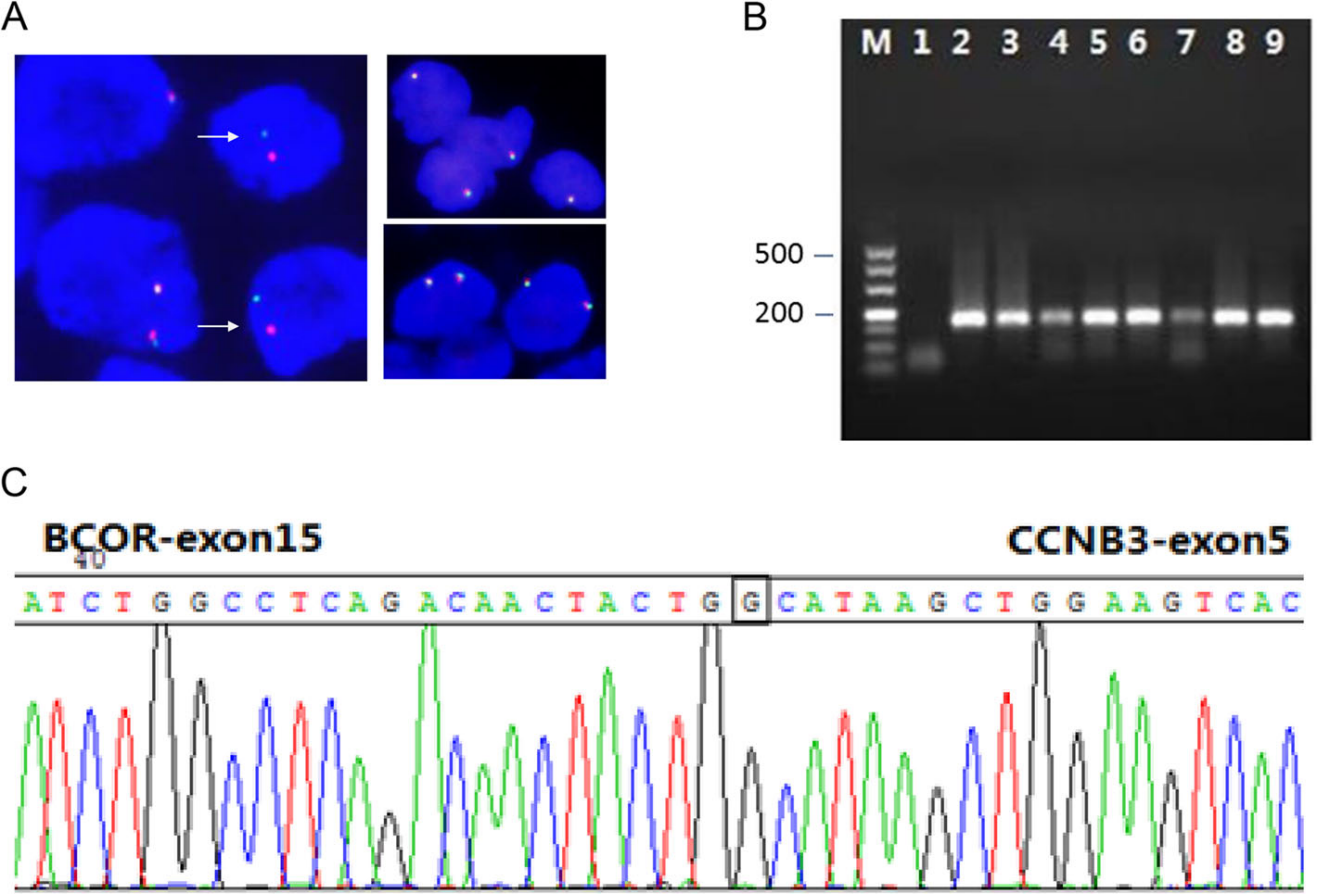
Genomic rearrangement in BCS. **A** Detection of *BCOR* gene rearrangement by fluorescence in situ hybridization, split signals of the BCOR gene (left, white arrows), normal signals of the BCOR gene (right up, male; right down, female). **B** Identification of the *BCOR-CCNB3* fusion transcripts by RT-PCR (M: marker; 1: negative control; 2: positive control; 3–9: case1–6, 8). **C** Schematic of the genomic breakpoint sequence in a representative case (Case 3). Original total magnification × 1000 (**A**)

Seven of the 8 cases carrying *BCOR* gene rearrangement by FISH were confirmed to have the *BCOR-CCNB3* fusion transcript by RT-PCR. The chimeric transcript joined the exon 15 of *BCOR* to exon 5 of *CCNB3* in all of the positive cases. The remaining 15 cases were negative (Fig. 4b, c).

### Treatment and follow-up

All 8 cases had clinical follow-up data. The average follow-up duration of the study group was 38 months (range from 7 to 46 months). All patients with available follow-up presented with localized disease at diagnosis. During the follow-up period, case 1 developed local recurrence and distant metastases to lung. Of the 8 patients, 5 cases (case 1, 2, 4, 5 and 8) received neoadjuvant chemotherapy followed by surgery and chemotherapy. Case 3 received chemotherapy and radiation therapy without surgery. Case 6 was receiving chemotherapy after surgery when the article was written and case 7 was receiving neoadjuvant chemotherapy meanwhile. The chemotherapy regimens used and the dose of radiation therapy adopted were listed in Table 1. Four cases showed significant response to the chemotherapy (> 90% necrosis/fibrosis) in the resection specimens (case 2, 4, 5 and 8). The patient (case 1) underwent curettage then the tumor relapsed 3 months later and amputation was adopted, but unfortunately the tumor metastasized to the lung and the patient died 2 years later. Five patients are alive in sustained complete remission (case 2, 3, 4, 5 and 8) after follow-up up to 42 months.

## Discussion

In this study, we investigated a series of 23 cases of *EWSR1* rearrangement-negative undifferentiated small round cell sarcoma and found 8 cases harboring the *BCOR* gene rearrangement. *BCOR-CCNB3* fusion transcript was detected in seven of 8 cases by RT-PCR. The 8 patients with *BCOR* gene rearrangement have a strong male predominance (M:F = 6:2) and predilection for children and adolescents. All tumors were located in lower extremities. The tumor locations were more common in bone (*n* = 6) than deep soft tissues (*n* = 2). Most of the cases showed predominantly monomorphic ovoid to short spindle cells arranged in intersecting fascicles, reminiscent of synovial sarcoma, with a rich capillary network. Some hypocellular areas were seen with myxoid stroma, consistent with previous reports [1, 4–8]. Since synovial sarcoma, solitary fibrous tumor, malignant peripheral nerve sheath tumor and osteosarcoma are among the potential differential diagnoses of *BCOR*-rearranged sarcomas, the detection of *BCOR* gene rearrangement is very important in the diagnostic appraisal of this lesion, particularly in needle core biopsies [9].

BCS as a recently defined genetic entity tumor among undifferentiated small round cell sarcoma. Most of the cases reported in articles were reappraised through a variety of molecular methods and screening from retrospective studies [1, 4–6, 8]. The original diagnoses in some of cases were mis-classified as ES, Ewing-like sarcoma, synovial sarcoma, and small cell osteosarcoma. In our series, three cases (case 1, 2 and 3) were originally diagnosed as Ewing-like sarcoma. Three cases (case 4, 5 and 8) were originally diagnosed as small cell osteosarcoma and received an osteosarcoma chemotherapy protocol. Therefore, accurate detection of *BCOR* gene rearrangements and other rare translocations are vitally important for appropriate patient management.

The sensitivity and specificity of *CCNB3* immunohistochemistry has been discussed recently [1, 3, 4, 10, 11]. Matsuyama et al. [6] argued that the complete sensitivity of CCNB3 immunohistochemistry in some previous studies was based on the screening method using CCNB3 immunohistochemistry [4, 11]. CCNB3 was not always expressed in BCS in other studies, especially in post chemotherapeutic or metastatic tumors [3]. BCOR immunohistochemistry is a highly sensitive marker in identifying small round cell sarcomas with *BCOR* gene rearrangement [12], but another report suggested that BCOR is less specific than CCNB3 for the diagnosis of BCS [6].

Our data showed high sensitivity of TLE1 expression for BCS (8/8, 100%). However, TLE1 expression was by no means specific for BCS, being present in Ewing-like sarcoma (9/15, 60%), Ewing sarcoma (3/7, 43%), malignant peripheral nerve sheath tumors (2/2, 100%) and synovial sarcoma (4/5, 80%). Regard to the specificity of TLE1 expression as a diagnostic maker for synovial sarcoma, published studies of TLE1 expression have shown conflicting results [13, 14]. Foo et al. have shown TLE1 protein expression to be a sensitive and specific marker for synovial sarcomas and can be used to distinguish poorly differentiated synovial sarcoma from histologic mimics [13]. However, Kosemehmetoglu et al. revealed that TLE1 was not only expressed in synovial sarcoma. TLE1 expression was also seen in 53 of 143 (37%) non-synovial sarcoma, such as malignant peripheral nerve sheath tumors, neurofibromas and schwannomas [14].

The sensitivity and specificity of the antibody may be related to the conditions such as tissue fixation, the dilutions of the antibody, the quality and sensitivity of the antibodies themselves as well as the IHC scoring method. Therefore, immunohistochemistry of CCNB3 and BCOR expression may not be sufficient for diagnosis of *BCOR*-rearranged sarcomas.

In this study, we show that FISH using dual color *BCOR* break-apart probe is a reliable assay. Because the *BCOR-CCNB3* fusion is caused by a paracentric inversion of 2 closely located genes *BCOR* and *CCNB3* on the short arm of chromosome X, it was thought that the two genes were too close (only 10 Mb apart) to be reliably detected by dual color break-apart probes. Therefore, FISH using the 3 color *BCOR-CCNB3* fusion assay has been advocated [2]. Our data shows that dual color *BCOR* break-apart probe could be suitable for the detection of *BCOR* gene rearrangement. In Matsuyama’s report [6], eight of the 9 cases were confirmed to have *BCOR* gene rearrangement using dual color *BCOR* break apart probe.

RT-PCR is a reliable assay to detect *BCOR-CCNB3* fusion transcript. The sensitivity and specificity of RT-PCR in our study are 87.5% (7/8) and 100% (15/15), respectively. In one case the *BCOR-CCNB3* fusion transcript was not detected by RT-PCR. The possible reason could relate to tumor cellularity as the percentage of the *BCOR* split cells was relatively low (21%) by the FISH assay. Another possible reason why RT-PCR was less than 100% sensitive is that *BCOR* may have other fusion partners besides *CCNB3,* such as *BCOR-MAML3* and *KMT2D-BCOR* [2, 15]. In additional to the fusion transcripts, BCOR internal tandem duplications have been identified [2].

As BCS is rare there is limited clinical outcome data. These tumors were originally classified among ES family of tumors, and as such have been managed with ES-related chemotherapy protocols [2, 16]. Three previous studies have suggested that BCS are chemoresponsive [2, 4, 7]. Cohen-Gogo et al. [7] showed a good histologic response (> 90% necrosis) in 83% (10/12) of the evaluable patients treated mainly with ES chemotherapy. Four of the 6 post chemotherapy resections showed complete response, whereas the remaining 2 had scattered residual tumor cells in Puls’s study [4]. Kao, et al. demonstrated 5 of the 9 patients were good response to chemotherapy with > 90% necrosis and 2 of the 9 patients with 60–90% necrosis [2]. In our study, 4 cases received induced chemotherapy showed a good histologic response (> 90% necrosis). However, 3 patients treated with chemotherapy before surgery were based on protocols for osteosarcoma, and 1 patient treated with protocols for ES. As both the osteosarcoma-based and the ES-based regimens were combination regimens, it is difficult to know which regimen was the one responsible for the definitive response. Controlled prospective studies will be necessary to choose an optimum therapy for BCS.

This study shows that the combination of FISH and RT-PCR to detect *BCOR* gene rearrangements are reliable assays and should be considered in the diagnostic workup of undifferentiated round cell tumors that are negative for the *EWSR1* gene rearrangement.

## Supplementary Information

**Additional file 1:** The summary of 38 cases detected by FISH with BCOR break apart probe.

## Data Availability

The datasets used and/or analysed during the current study are available from the corresponding authors on reasonable request.

## Abbreviations

BCS: *BCOR-CCNB3* sarcoma
IHC: Immunohistochemistry
FISH: Fluorescence in situ hybridization
RT: Reverse Transcription
PCR: Polymerase Chain Reaction
ES: Ewing sarcoma
