# Supplementary material for: Detection of BCOR gene rearrangement in Ewing-like sarcoma: an important diagnostic tool
Source: Diagn Pathol. 2021 Jun 8;16:50. doi: 10.1186/s13000-021-01114-2 (PMC8185946; doi:10.1186/s13000-021-01114-2)
Supplement: Supplementary file 1 — Additional file 1: The summary of 38 cases detected by FISH with BCOR break apart probe. [file 13000_2021_1114_MOESM1_ESM.pdf]

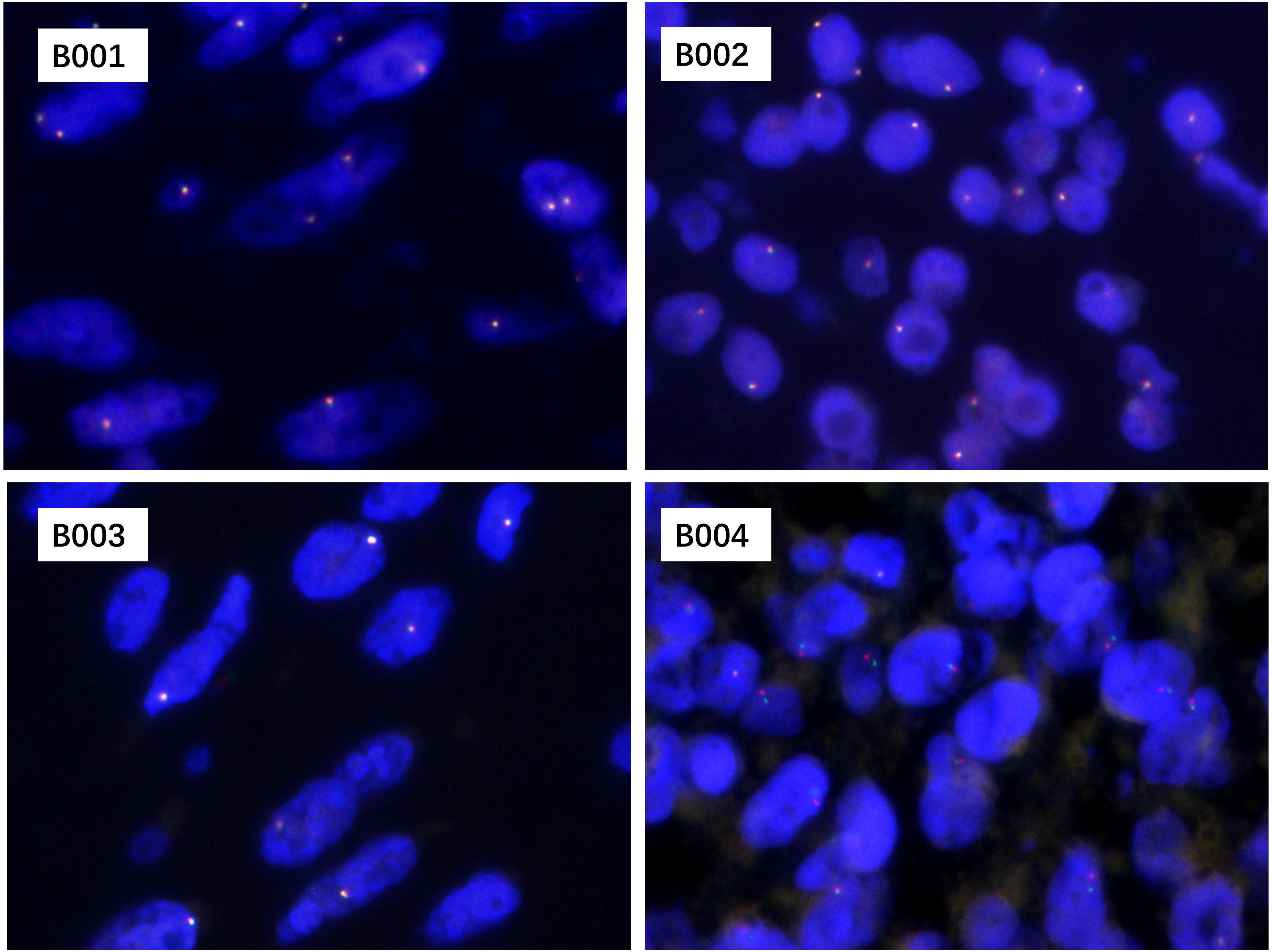
B001
B002
B003
B004

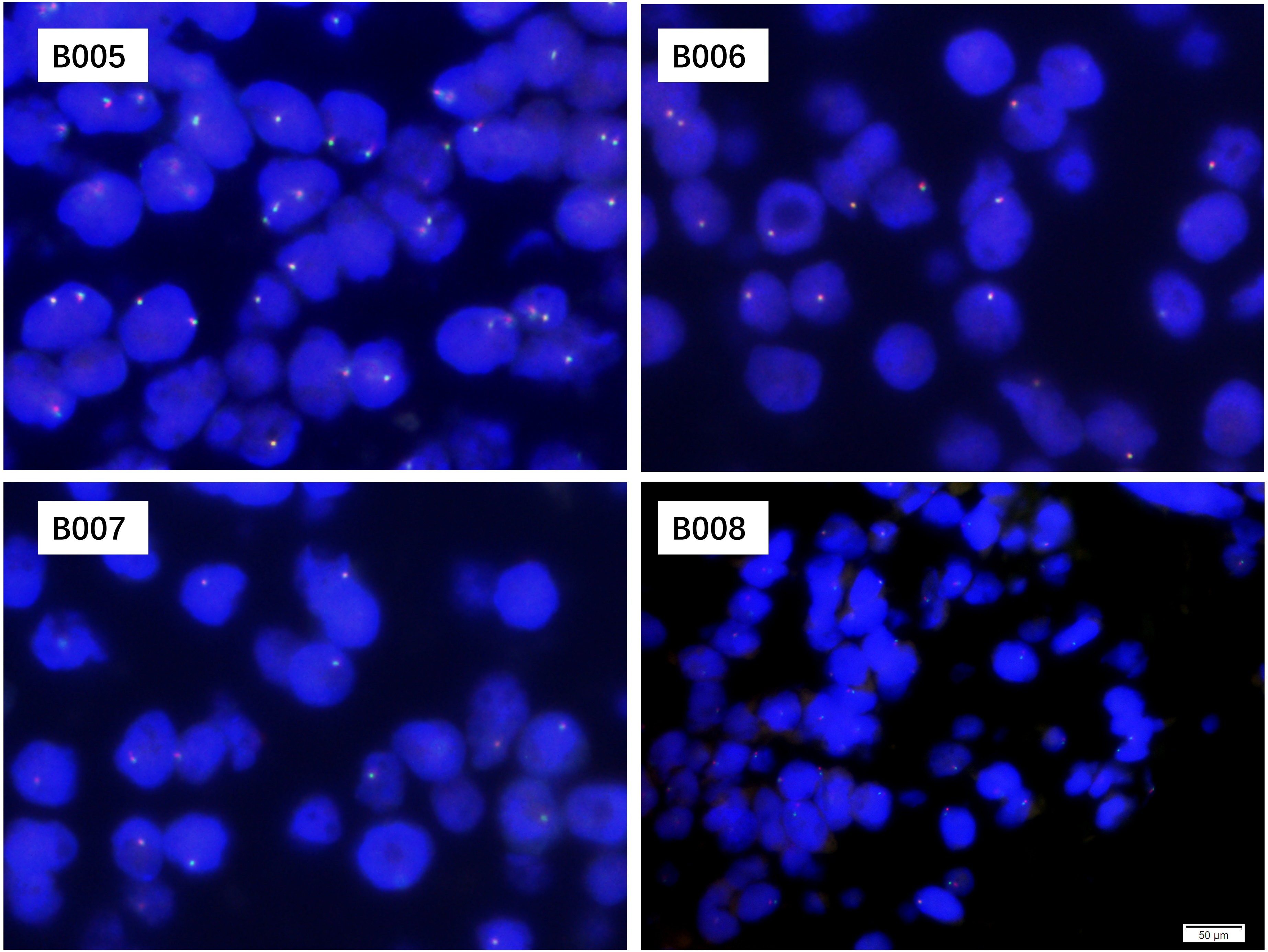
B005
B006
B007
B008
50 μm

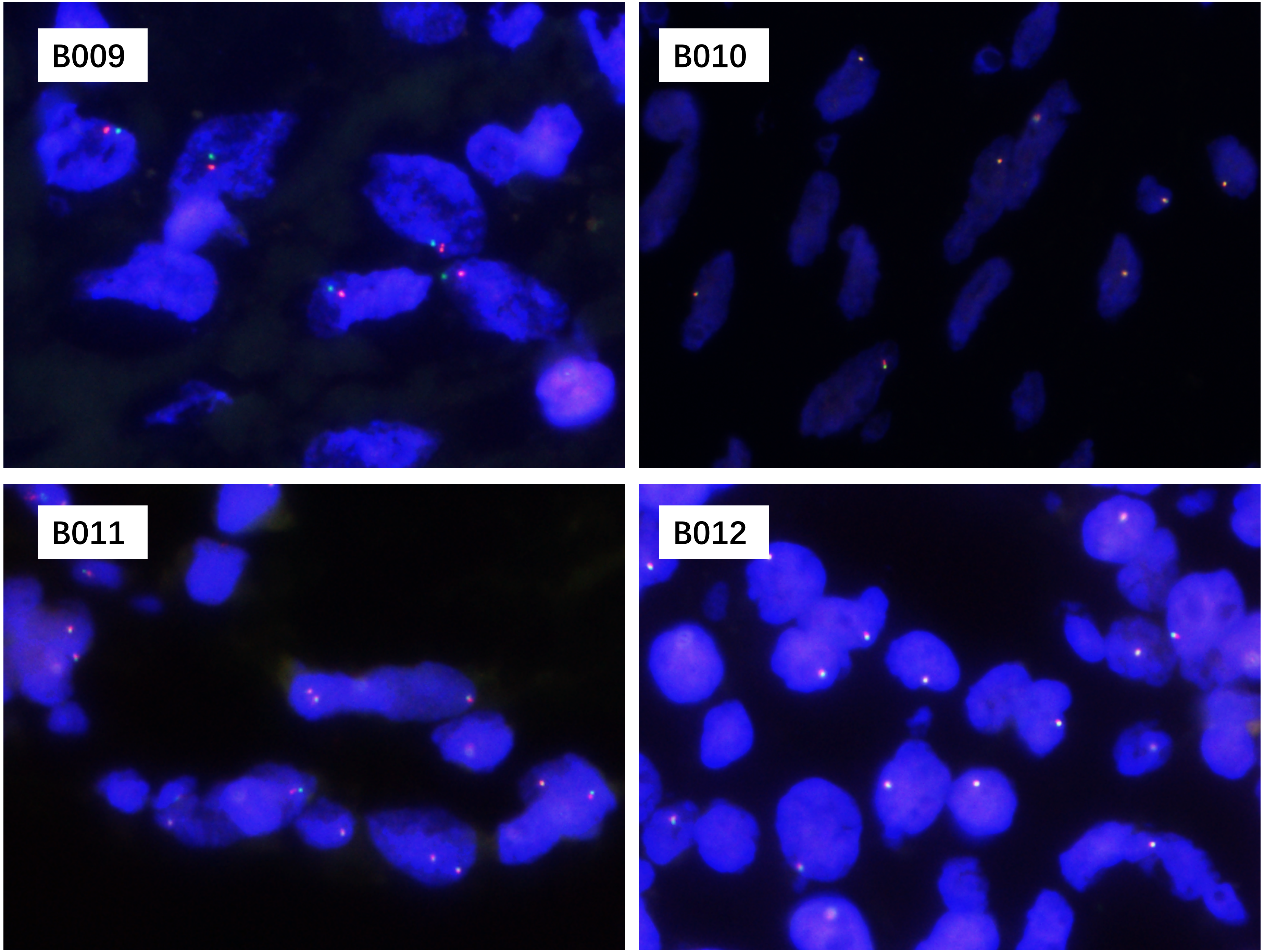
B009
B010
B011
B012

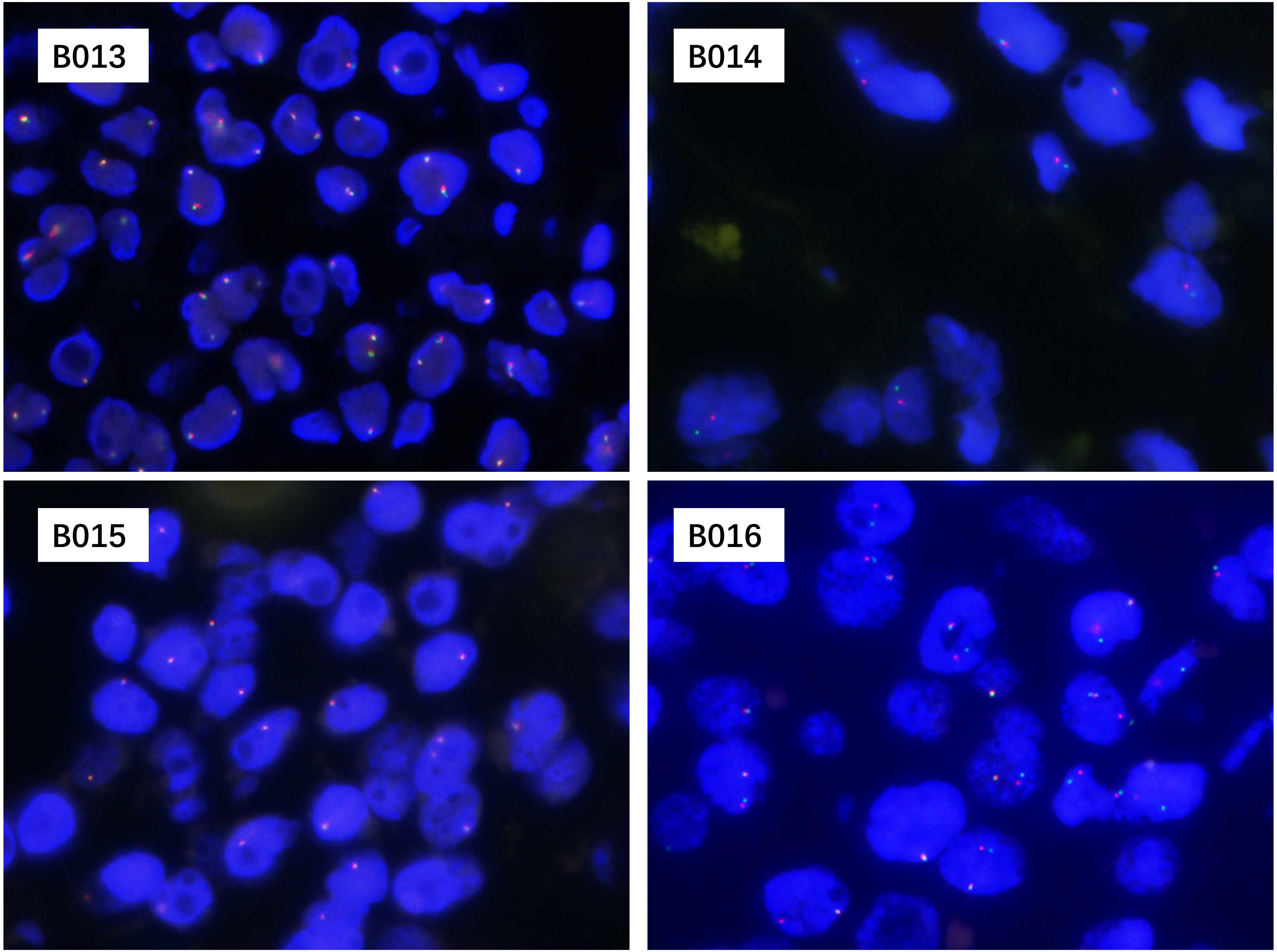
B013
B014
B015
B016

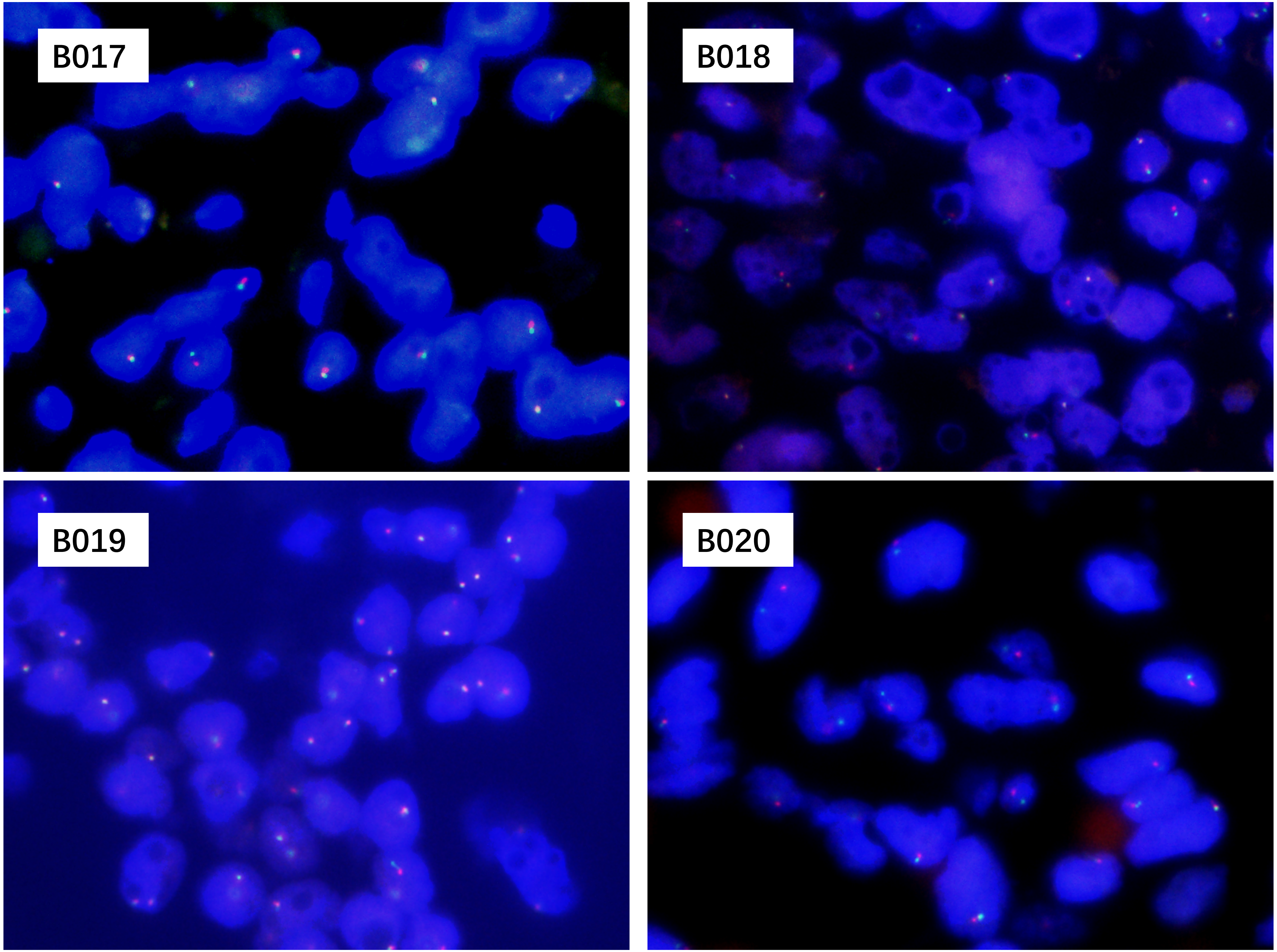

B017
B018
B019
B020

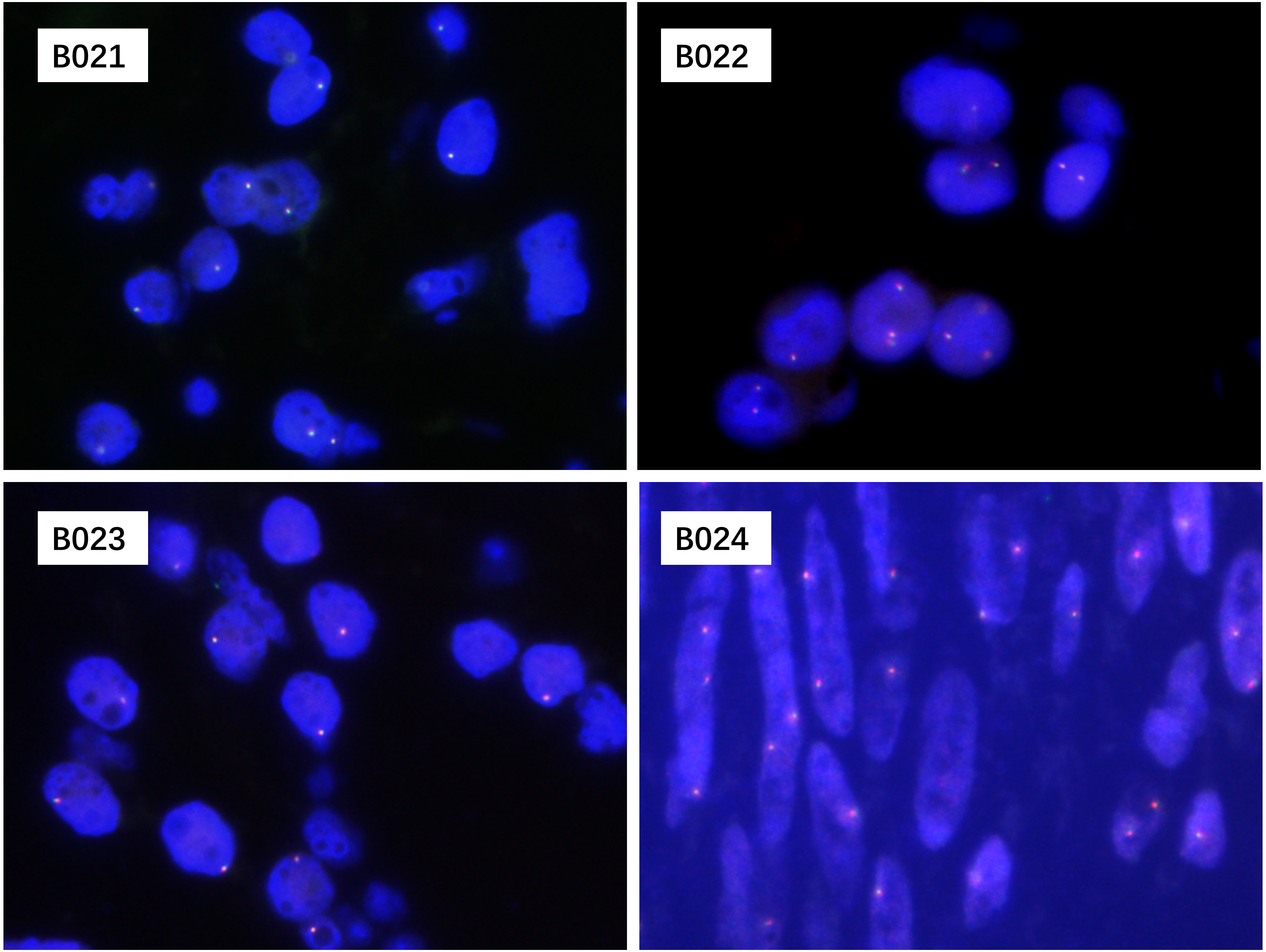
B021
B022
B023
B024

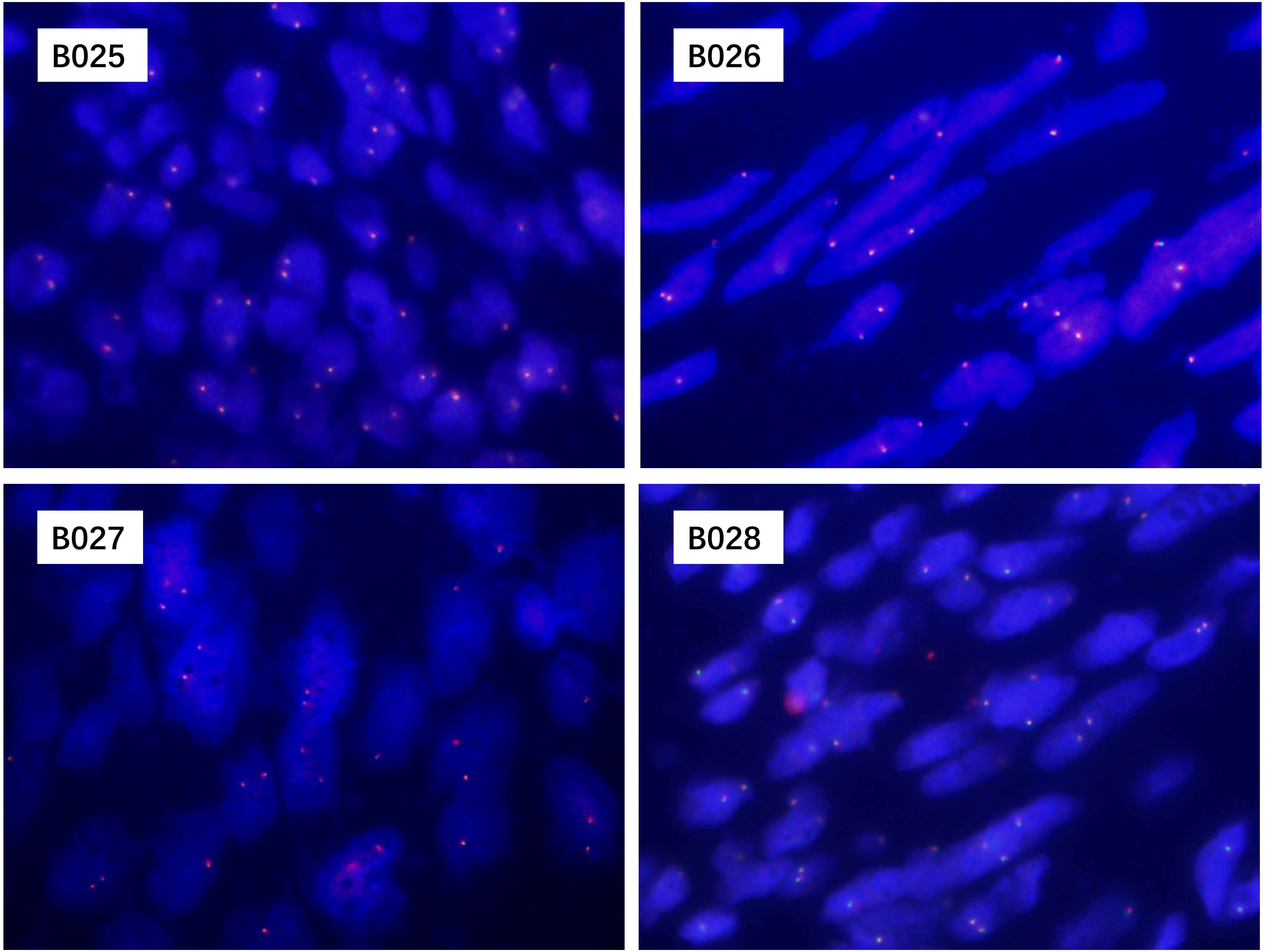
B025
B026
B027
B028

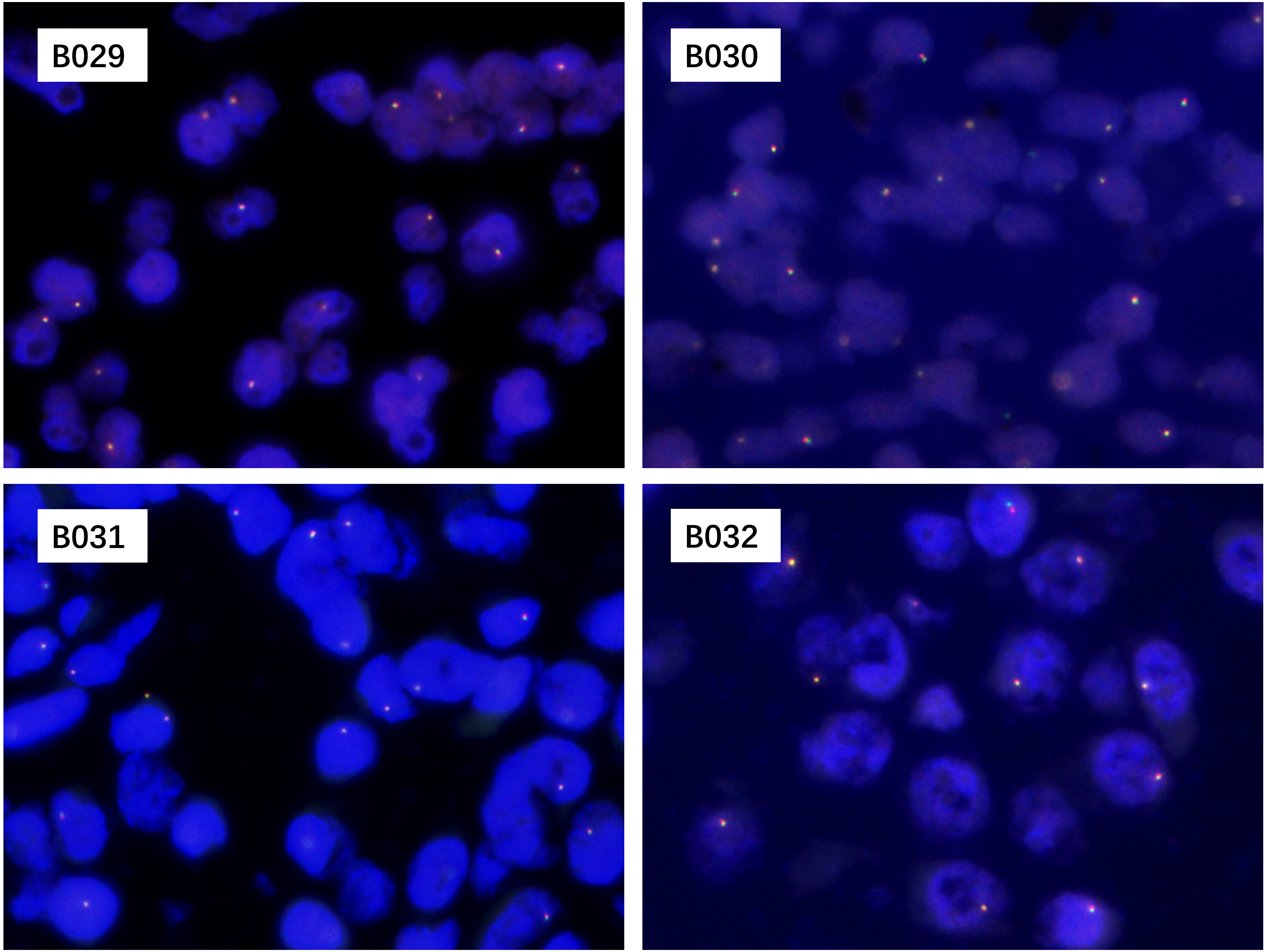
B029
B030
B031
B032

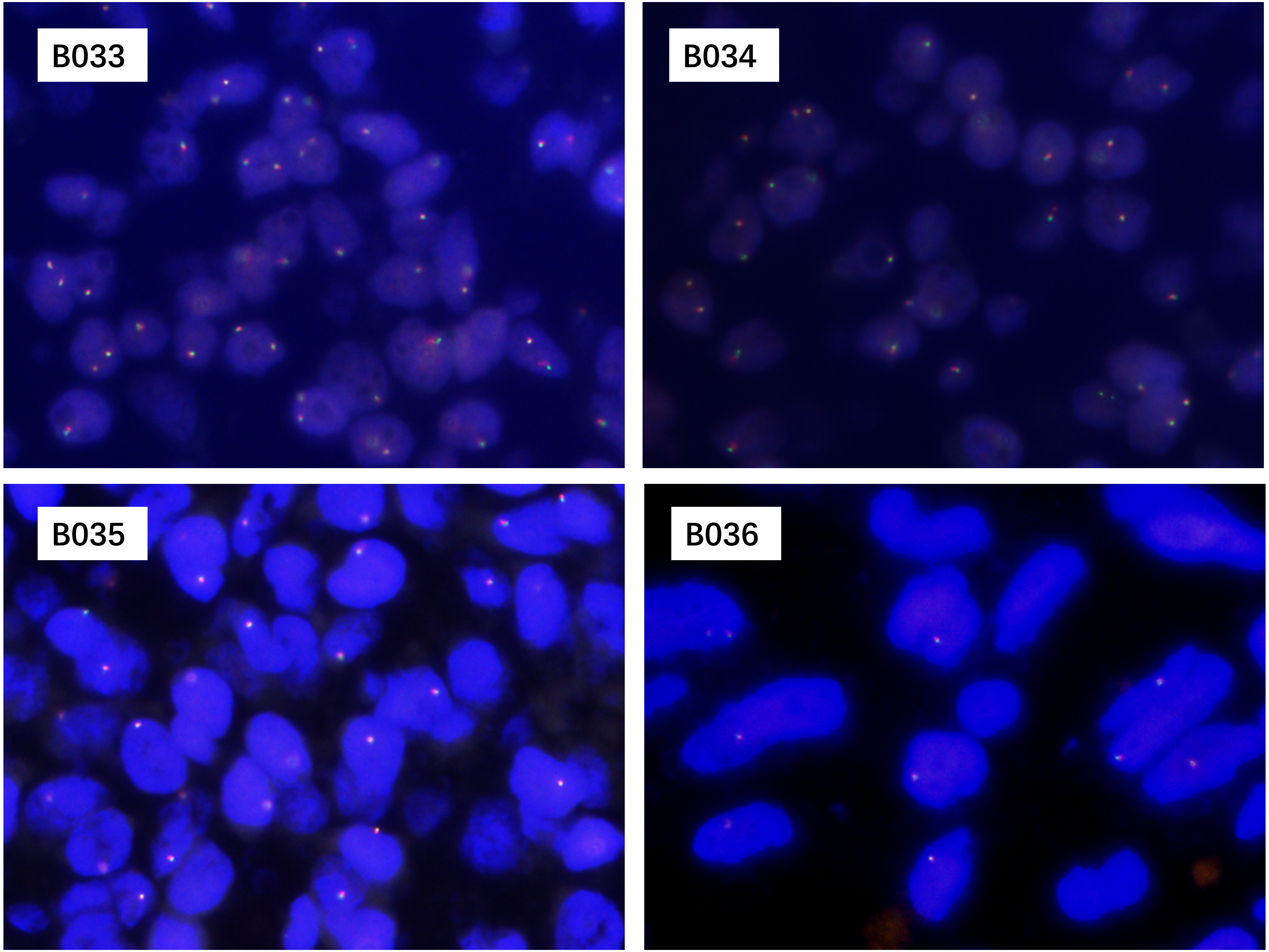
B033
B034
B035
B036

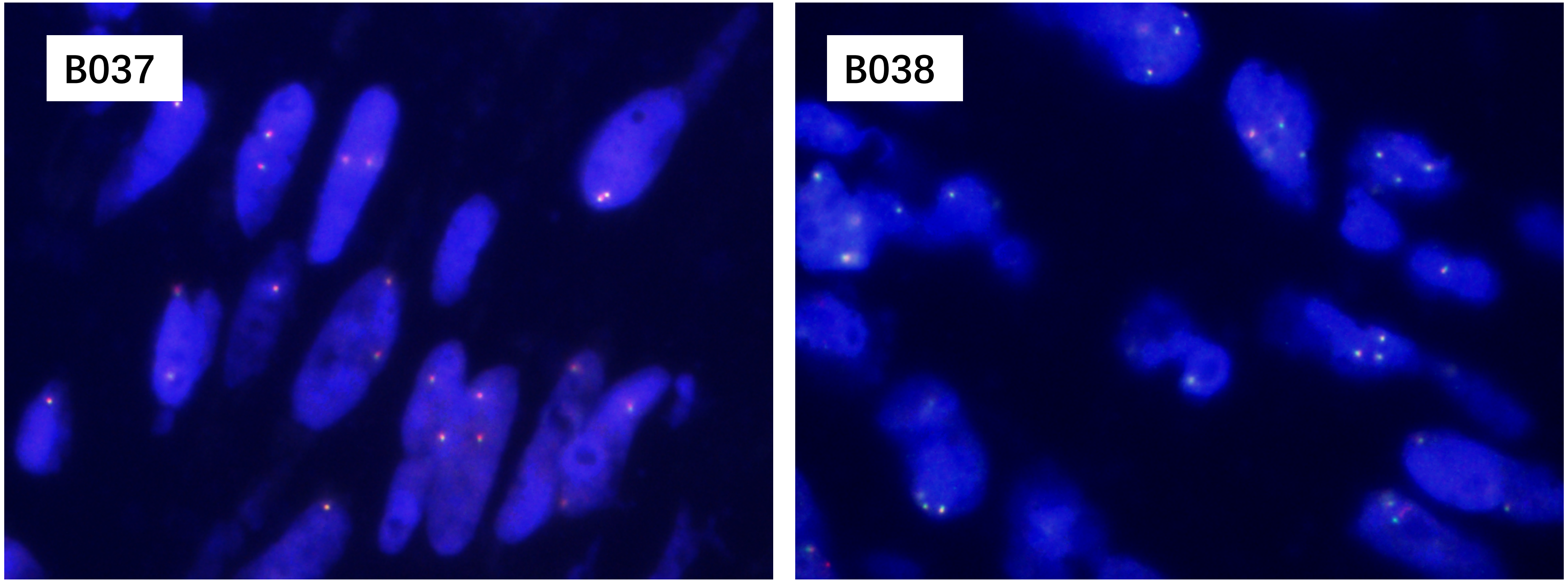
B037
B038

.

Supplementary Figure 1. 38 cases detected by FISH with BCOR break apart probe

1. B001: ELS, case 1, *BCOR* gene rearrangement negative
2. B002: ELS, case 2, *BCOR* gene rearrangement negative
3. B003: ELS, case 3, *BCOR* gene rearrangement negative
4. B004: BCS, Case 3, *BCOR* gene rearrangement positive
5. B005: ELS, case 4, *BCOR* gene rearrangement negative
6. B006: ELS, case 5, *BCOR* gene rearrangement negative
7. B007: ELS, case 6, *BCOR* gene rearrangement negative
8. B008: BCS, Case 2, *BCOR* gene rearrangement positive
9. B009: BCS, Case 1, *BCOR* gene rearrangement positive
10. B010: ELS, case 7, *BCOR* gene rearrangement negative
11. B011: ELS, case 8, *BCOR* gene rearrangement negative
12. B012: ELS, case 9, *BCOR* gene rearrangement negative

13: B013: ELS, case 10, *BCOR* gene rearrangement negative

14. B014: BCS, Case 4, *BCOR* gene rearrangement positive

15. B015: ELS, case 11, *BCOR* gene rearrangement negative

16. B016: BCS, Case 6, *BCOR* gene rearrangement positive

17. B017: BCS, Case 7, *BCOR* gene rearrangement positive

18. B018: BCS, Case 8, *BCOR* gene rearrangement positive

19. B019: ELS, case 12, *BCOR* gene rearrangement negative

20. B020: BCS, Case 5, *BCOR* gene rearrangement positive

21. B021: ELS, case 13, *BCOR* gene rearrangement negative

22. B022: ELS, case 14, *BCOR* gene rearrangement negative

23. B023: ELS, case 15, *BCOR* gene rearrangement negative

24. B024: Negative control, case 1 (SS), *BCOR* gene rearrangement negative

25. B025: Negative control, case 2 (SS), *BCOR* gene rearrangement negative

26. B026 Negative control, case 3 (SS), *BCOR* gene rearrangement negative

27. B027: Negative control, case 4 (SS), *BCOR* gene rearrangement negative

28. B028: Negative control, case 5 (SS), *BCOR* gene rearrangement negative

29. B029: Negative control, case 6 (EWS), *BCOR* gene rearrangement negative

30. B030: Negative control, case 7 (EWS), *BCOR* gene rearrangement negative

31. B031: Negative control, case 8 (EWS), *BCOR* gene rearrangement negative

32. B032: Negative control, case 9 (EWS), *BCOR* gene rearrangement negative

33. B033: Negative control, case 10 (EWS), *BCOR* gene rearrangement negative

34. B034 Negative control, case 11 (EWS), *BCOR* gene rearrangement negative

35. B035: Negative control, case 12 (EWS), *BCOR* gene rearrangement negative

36. B036: Negative control, case 13 (OS), *BCOR* gene rearrangement negative

37. B037: Negative control, case 14 (MPNST), *BCOR* gene rearrangement negative

38. B038 Negative control, case 15 (MPNST), *BCOR* gene rearrangement negative

(ELS, Ewing-like sarcoma; BCS, BCOR-CCNB3 sarcoma; SS, synovial sarcoma; EWS, Ewing sarcoma; OS, osteosarcoma; MPNST, malignant peripheral nerve sheath tumor)
